# Faustoviruses: Comparative Genomics of New *Megavirales* Family Members

**DOI:** 10.3389/fmicb.2016.00003

**Published:** 2016-02-05

**Authors:** Samia Benamar, Dorine G. I. Reteno, Victor Bandaly, Noémie Labas, Didier Raoult, Bernard La Scola

**Affiliations:** ^1^UM63 CNRS 7278 IRD 198 INSERM U1095, Unité de Recherche sur les Maladies Infectieuses et Tropicales Emergentes, Facultés de Médecine et de Pharmacie, Aix-Marseille UniversityMarseille, France; ^2^Pôle des Maladies Infectieuses et Tropicales Clinique et Biologique, Fédération de Bactériologie-Hygiène-Virologie, Institut Hospitalo-Universitaire Méditerranée Infection, Centre Hospitalo-Universitaire Timone, Assistance Publique - Hôpitaux de MarseilleMarseille, France

**Keywords:** Faustovirus, giant viruses, pan-genome, phylogenetic, genomic comparison

## Abstract

An emerging interest for the giant virus discovery process, genome sequencing and analysis has allowed an expansion of the number of known *Megavirales* members. Using the protist *Vermamoeba* sp. as cell support, a new giant virus named Faustovirus has been isolated. In this study, we describe the genome sequences of nine Faustoviruses and build a genomic comparison in order to have a comprehensive overview of genomic composition and diversity among this new virus family. The average sequence length of these viruses is 467,592.44 bp (ranging from 455,803 to 491,024 bp), making them the fourth largest *Megavirales* genome after Mimiviruses, Pandoraviruses, and *Pithovirus sibericum*. Faustovirus genomes displayed an average G+C content of 37.14 % (ranging from 36.22 to 39.59%) which is close to the G+C content range of the *Asfarviridae* genomes (38%). The proportion of best matches and the phylogenetic analysis suggest a shared origin with *Asfarviridae* without belonging to the same family. The core-gene-based phylogeny of Faustoviruses study has identified four lineages. These results were confirmed by the analysis of amino acids and COGs category distribution. The diversity of the gene composition of these lineages is mainly explained by gene deletion or acquisition and some exceptions for gene duplications. The high proportion of best matches from Bacteria and *Phycodnaviridae* on the pan-genome and unique genes may be explained by an interaction occurring after the separation of the lineages. The Faustovirus core-genome appears to consolidate the surrounding of 207 genes whereas the pan-genome is described as an open pan-genome, its enrichment via the discovery of new Faustoviruses is required to better seize all the genomic diversity of this family.

## Introduction

Viruses infecting the Eukaryota cell including the *Phycodnaviridae*, *Poxviridae*, *Iridoviridae*, *Ascoviridae*, *Asfarviridae*, *Mimiviridae*, and *Marseilleviridae* families are representative members of the *Megavirales* order families (Colson et al., 2012, 2013). Since the discovery of the *Acanthamoeba polyphaga mimivirus* (La Scola et al., 2003; Raoult et al., 2004; Iyer et al., 2006; Raoult and Forterre, 2008; Yutin et al., 2009, 2014; Yutin and Koonin, 2012), the first described protist-associated virus, other giant viruses have been nearly all isolated from *Acanthamoeba castellanii* and *A.* *polyphaga* (Pagnier et al., 2013; Philippe et al., 2013; Legendre et al., 2014; Antwerpen et al., 2015; Scheid, 2015), probably neglecting a wide part of the giant virus world. Recently, Reteno et al. (2015) using the protist *Vermamoeba* sp. as cell support, have isolated a new giant virus named Faustovirus. It was proposed to be the first member of the eighth family in the *Megavirales* order. The Faustoviruses were shown to share several features with *Asfarviridae*. Phylogenetic analyses indicate that the evolutionary distance between Faustovirus and Asfaviruses is comparable to that between Pandoraviruses and Phycodnaviruses (Reteno et al., 2015). In this study, we describe the genome sequences of nine Faustoviruses and build a genomics comparison in order to have a comprehensive overview of genomic composition and diversity among this new virus family. Aspects of the pan-genome were described and discussed. A phylogenetic analysis with all the core-genes allowed us to identify four lineages. We observed that despite the divergent features, Faustoviruses show a convergent adaptation to their intracellular life.

### Faustovirus Isolates

Isolation of Faustovirus strain E12 was previously described in detail (Reteno et al., 2015). The other eight strains analyzed herein were isolated using the same strategy in sewage samples. Faustovirus E23, E24 and E9 were isolated from Marseille sewage samples, France. Faustovirus D3, D6, D5a and D5b were isolated from Dakar sewage samples, Senegal. Faustovirus Liban was isolated from Tripoli El Mina sea water sample, Lebanon.

### Genome Sequencing, Data Assembly and General Features Prediction

Faustovirus E12 being available yet (Reteno et al., 2015), the other eight genomes were sequenced using MiSeq Technology (Illumina, Inc., San Diego, CA, USA) by using paired-end and mate-pair applications in parallel, in a 2- × 251-bp run for each bar-coded library. The reads were assembled *de novo* into a contigs using Mira 3.4. SSPACE software v1.0 combined to GapFiller were also used to enhance the assembly (Boetzer et al., 2011; Nadalin et al., 2011), remaining gaps were closed using Sanger sequencing of PCR products. Coding DNA Sequences (CDSs) were predicted using Prodigal (Hyatt et al., 2009). Data were submitted to the EMBL database and was assigned Bio-projects numbers (E24: PRJNA279158, D5a: PRJNA279159, D5b: PRJNA279160, D3: PRJNA279161, D6: PRJNA279164, Liban: PRJNA279165, E9: PRJNA279166, E23: PRJNA279157).

### Comparative Genomics

A comparative analysis of the nine genomes was generated by creating a reference database of all the protein sequences. COGtriangles and OrthoMCL clustering algorithms were used to create protein clusters. Pangenome and core-genes was defined by using GET_HOMOLOGUES with the following parameters: 75% as minimum coverage, 30% as minimum identity in Blastp pairwise alignments and 1e-05 as maximum *E*-value.

### Phylogenetic Tree Construction

Protein sequences were aligned using the MUSCLE program with default parameters; All the alignment were conserved even the columns containing a large fraction of gaps. A preliminary maximum-likelihood tree was constructed using the FastTree program with default parameters (JTT evolutionary model, discrete gamma model with 20 rate categories).

### Genomics of Faustoviruses

**FIGURE 1:**
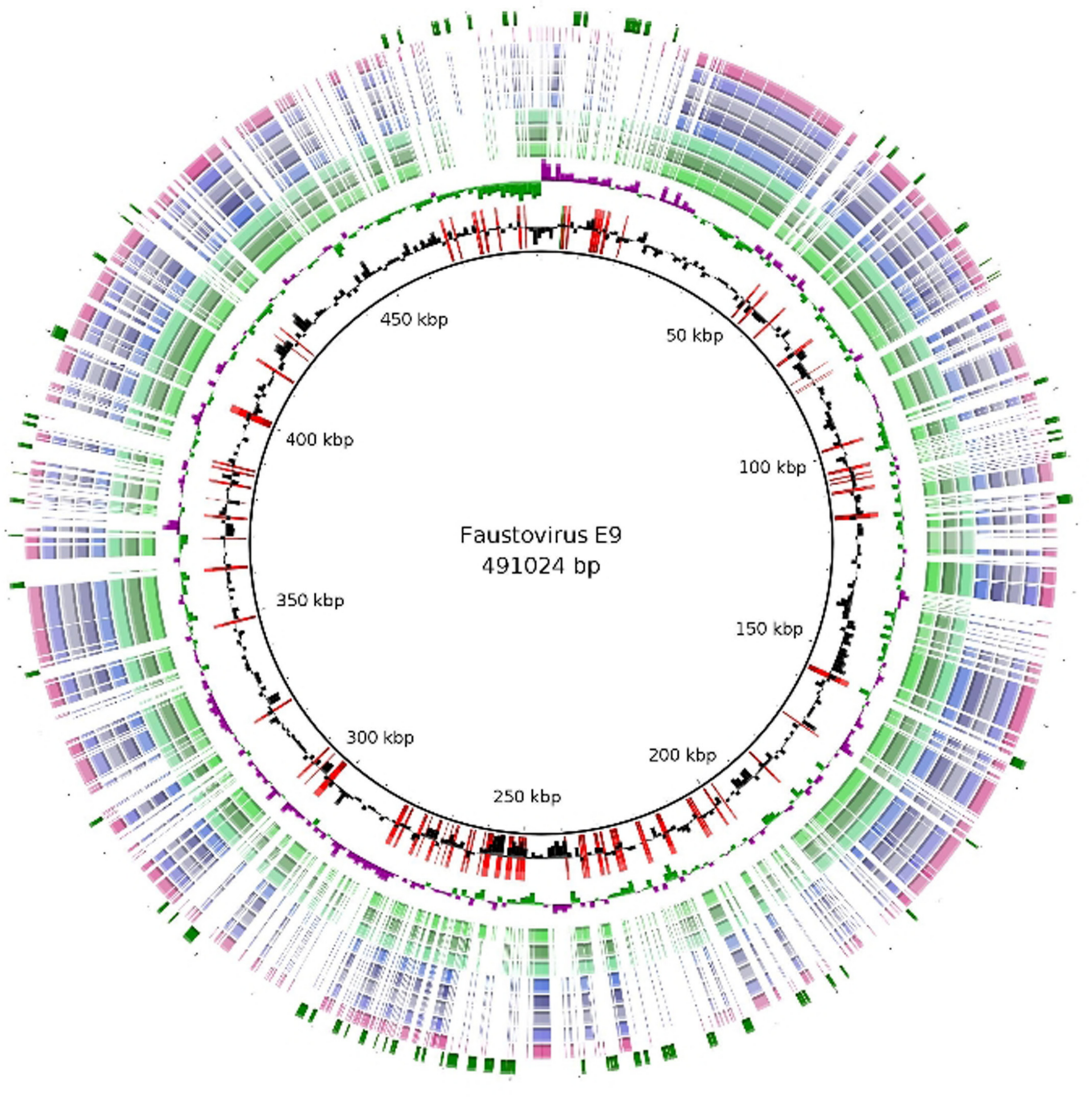
**Regions of variability among the Faustovirus genomes**. BLAST based genome showing regions of variability of Faustovirus E9 and the other Faustoviruses. From outer to inner circle: Unique genes of Faustovirus E9, Faustovirus Liban, Faustovirus D5a, Faustovirus E24, Faustovirus E23, Faustovirus E12, Faustovirus D5b, Faustovirus D6, Faustovirus D3, GC Skew and GC content. The unique genes of Faustovirus E9 were shown in the first circle (Green) and superimposed (in red) on the GC content circle.

The genome features of the nine Faustoviruses are summarized in **Table 1**. The average sequence length is 467,592 bp, making them the fourth largest *Megavirales* genome after Mimiviruses, Pandoraviruses and *P. sibericum* (Arslan et al., 2011; Philippe et al., 2013; Antwerpen et al., 2015; Scheid, 2015). These viruses have circular genomes except for Faustovirus Liban, for which genome could not be closed suggesting linear genome (**Figure 1**). Faustovirus D3 represents the strain with the smallest genome (with a size of 455,803 bp), while Faustovirus E9 possessed the largest genome (with a size of 491,024 bp). Faustovirus genomes displayed an average G+C content of 37.14% (ranging from 36.22 to 39.59%). No correlation was observed between the genome size and the GC content.

**Table 1:** General features of nine complete Faustovirus genomes.

	Faustovirus E12	Faustovirus E23	Faustovirus E24	Faustovirus D5a	Faustovirus D5b	Faustovirus D3	Faustovirus D6	Faustovirus E9	Faustovirus Liban
Genome length (bp)	466,265	465,956	466,012	466,051	464,523	455,803	462,011	491,024	470,687
GC content (%)	36.22	36.22	36.22	36.21	37.66	37.76	37.67	39.59	36.74
Number of genes	457	519	518	517	507	495	509	511	518
Hypothetical proteins	303	457	456	454	433	430	438	449	467

### Pan-Genome and Core-Genome Analysis

In order to have a coherent comparative analysis, the same software for the prediction of the Open Reading Frame (ORF) was used. In this fashion, a comparable percent of DNA coding was obtained for each genome except for Faustovirus E12 (**Table 1**). In fact analysis of this last isolate benefited of proteomic study allowing deleting forty three ORFans (ORFs with no detectable homolog) and small genes (>100 bp) whereas for other isolates only an *in silico* prediction of CDS was done.

**FIGURE 2:**
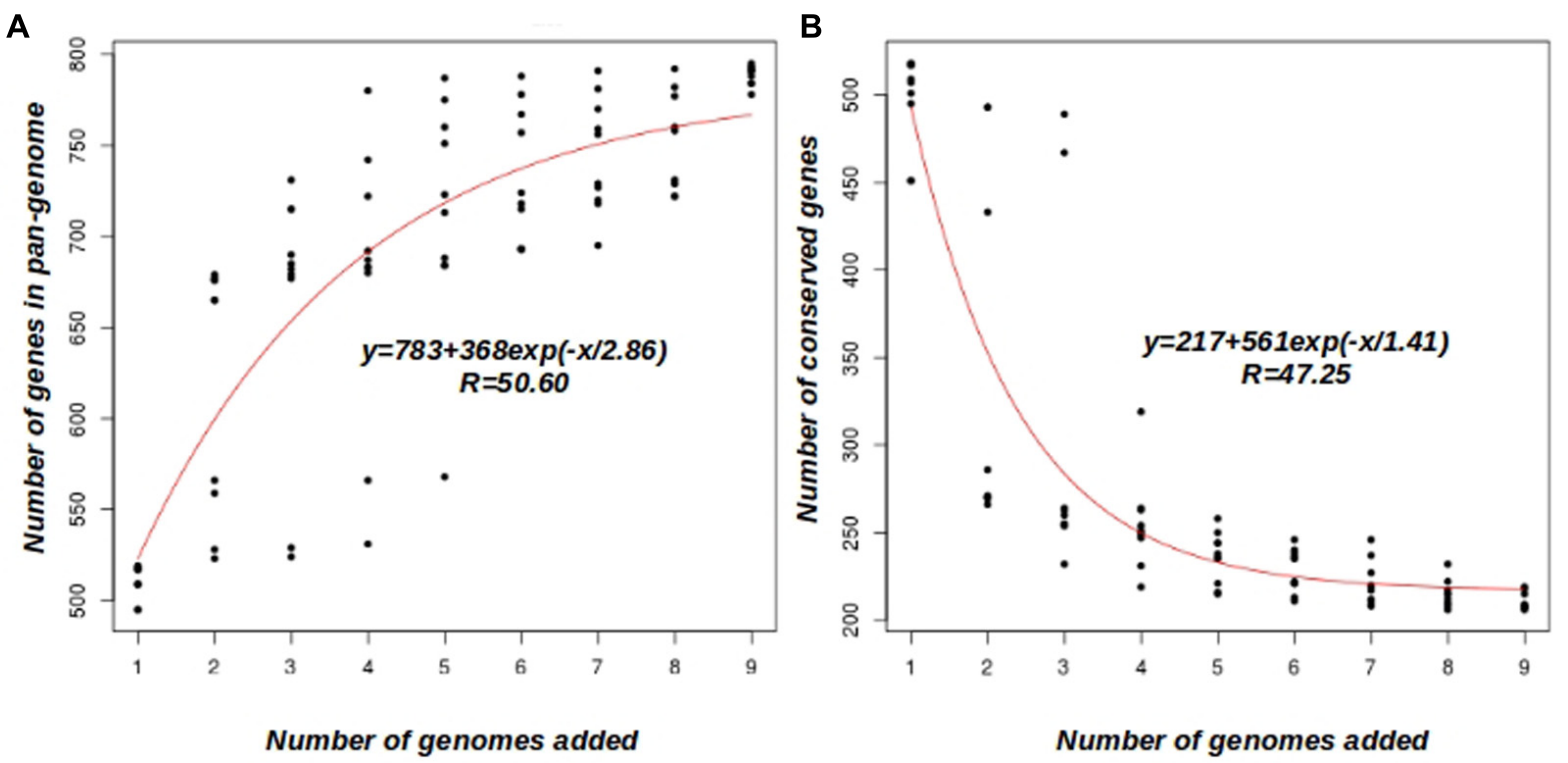
**Faustovirus pan-genome and core-genome. (A)** Accumulation curve for the total number of genes plotted against the number of genomes analyzed. **(B)** Accumulation curves for the number of genes in common plotted against the number of added genomes, the coregenes count 148 hepothetical protein (71.5%). The deduced mathematical function and the residual standard error are also reported.

A clustering analysis of the orthologous group of proteins based on COG triangles and OrthoMCL clustering algorithms, using the nine Faustovirus genomes, revealed the presence of 872 clusters. An overview of the Faustoviruses’ pan-genome appears not to have been reached, as shown in **Figure 2A**. The average number of new genes added by each new genome was 30, and this number decreased to 19 after the addition of five genomes. According to the deduced mathematical function, the Faustovirus pan-genome probably surpasses 1000 genes; this finding suggests that the Faustoviruses’ pan-genome is still open. On the other hand, the core-genome appears to reach a plateau at around 207 genes (**Figure 2B**). The curve indicates it will remain relatively constant, even if many more genomes are added. The number of core-genes in each genome is constant, except for Faustovirus Liban (five genes are in two copies per cluster), indicating no duplicated genes and an absence of paralogs. In the remaining clusters, 13 other duplications were observed (5 in Faustovirus E9 and 8 in Faustovirus Liban). These findings were confirmed by the Faustovirus genome pairwise dot plot (Supplementary Figure S4). Indeed, the dot plot showed a visible duplication in Faustovirus Liban. The set of 207 genes identified as core-genes comprises mainly orthologous groups of genes (COGs) of the NCVOGs, which are present in at least two viral families (Yutin and Koonin, 2009; Yutin et al., 2009). Indeed, 78 core-genes (37.68% of the core-genes) have a significant blast result against the NCVOG database (*E*-value <10e-3) and there are 148 hypothetical protein in the coregenes (71.5%).

The core-genome displays a larger number of genes with detectable homologs in the NCBI bank (Supplementary Figure S4) than the pan-genome (44.92 and 21.33% respectively). For almost half (53.76%) of the Faustovirus core-genes with detectable homologs, best matches were with genes from viruses (*Asfarviridae*, *Iridoviridae*, *Malacoherpesviridae*, *Marseilleviridae*, *Mimiviridae*, *Myoviridae*, and *Phycodnaviridae*). One hundred and fourteen genes (55.07% of the core-genes) are specific to the Faustoviruses and are not found in any other organism sequence, while 16 genes (7.72% of the core-genes) are specific to the Faustoviruses and the *Asfarviridae* (Supplementary Tables S1 and S2). The proportion of best matches from *Asfarviridae* is greater in the core-genome (74%) than in the pan-genome (61.25%), while a contrary effect was observed with the *Phycodnaviridae* more abundant in the pan-genome (20%) than in the core-genome (8%). No best match with genes from *Malacoherpesviridae* and *Marseilleviridae* was observed in the core-genome while 3 best matches were noticed in the pan-genome (1 from the *Malacoherpesviridae* and two from *Marseilleviridae*). On the whole, the pan-genome has fewer good matches with genes from viruses (43.01%) than the core-genome (Supplementary Figure S4). In contrast, we observed more best matches from Bacteria in the pan-genome (30.10%) than in the core-genome (22.58%). Best matches with genes from Eukaryota are in a similar proportion in the pan-genome (23.11%) and the core-genome (21.50%). Among the best matches from Eukaryota, five genes specific to *Viridiplantae* and several others found only in plants and Fungi were observed. Six best matches were with genes from Phytophthora (*Phytophthora parasitica:* 4, *Phytophthora sojae*: 1*, Phytophthora infestans T30-4*: 1) and were distributed equitably on the core and pan-genome (Supplementary Table S1). Seven best matches were with genes from Archaea including two with a known function (transcription factor S-II-related protein and putative DNA-directed RNA polymerase subunit D) identified in the core-genome.

### Faustovirus Classification

**FIGURE 3:**
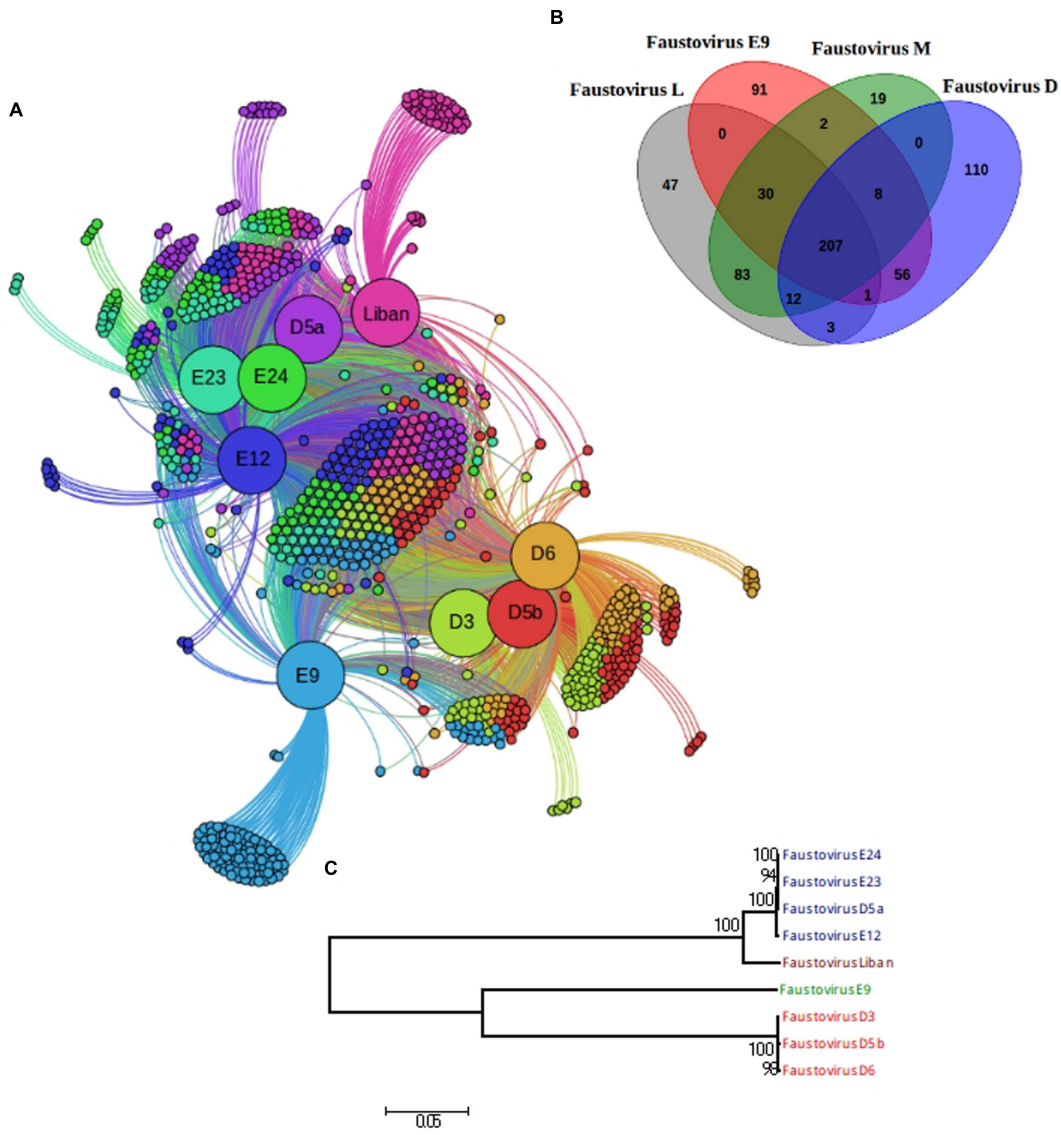
**Comparative genomics of fully sequenced Faustovirus genomes and core-gene-based phylogeny. (A)** Protein clusters and viruses’ connection, nodes correspond to protein clusters (small nodes) or viruses. Edges signify presence of a protein cluster in the virus. Each protein cluster is connected to the virus having a protein therein. Viruses and clusters are placed depending on the links between them. Coregenes are in the middle of the figure and the single genes on the suburbs. **(B)** Venn diagram representing the orthologous and unique gene families of the four lineages. **(C)** Phylogenetic tree based on the 207 core-genes. Maximum likelihood based on the phylogenetic analysis of a concatenated set of 207 genes.

In order to build a robust phylogenetic analysis we used the 207 core-genes, comprising in total 78,429 amino acid positions, to distinguish the different Faustovirus lineages (**Figure 3C**). The phylogenetic tree shows height robustness. Indeed, the nodes were supported by 100% of the bootstrap iterations, with a slightly lower supported proportion (94–98%) observed in two nodes. Based on this phylogenetic construction, four lineages were identified: lineage M comprises the founding member Faustovirus E12 with E24, E23 and D5a; lineage D is formed by Faustovirus D3, D6 and D5b; lineage L contains only Faustovirus Liban and E9. Lineage D is exclusively formed of viruses identified in the same sewage environment (Senegal), like lineages L and E9 (Lebanon and French respectively). In contrast, lineage M is formed of three viruses isolated in France and one in Senegal.

**FIGURE 4:**
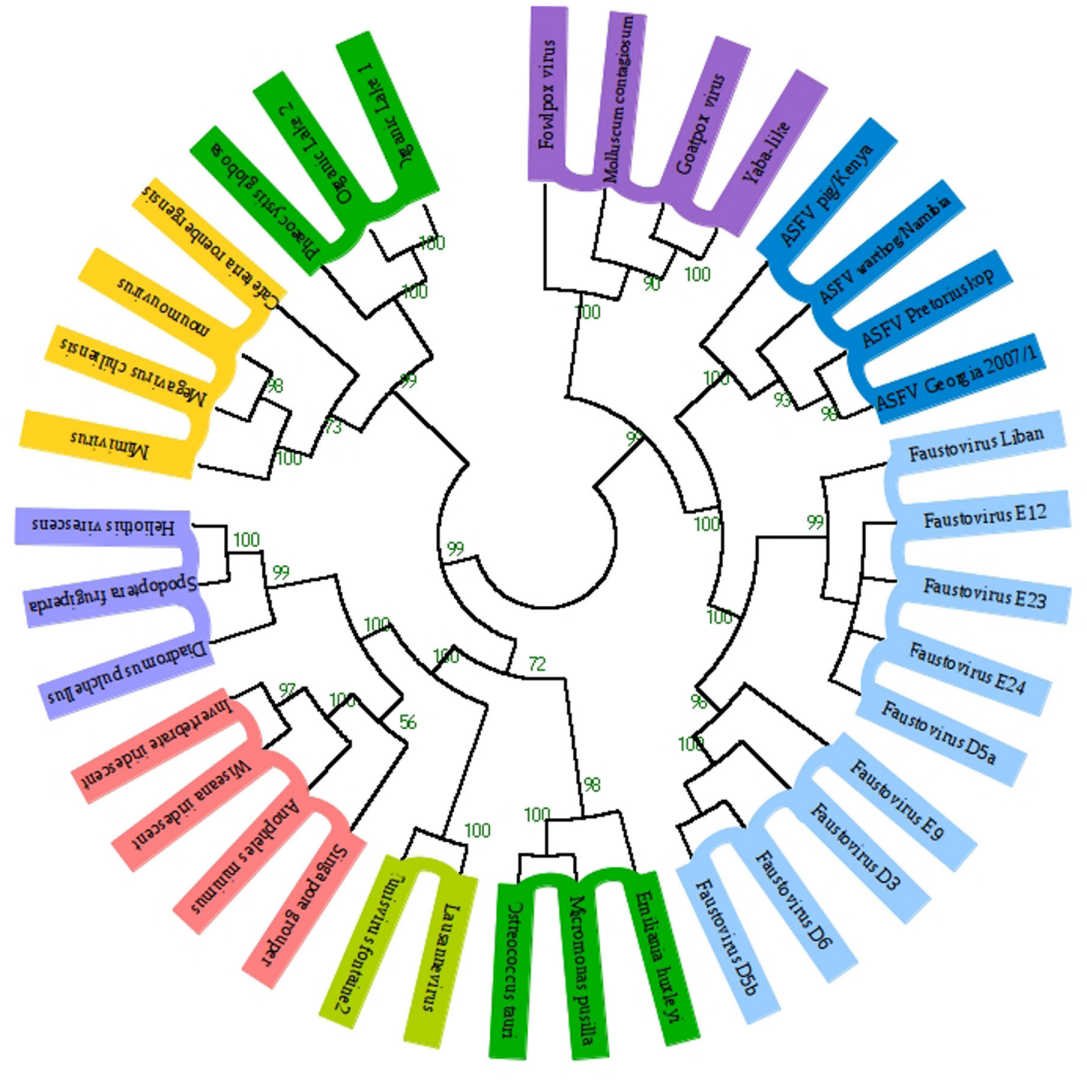
**Phylogenetic reconstruction based on concatenated A32-like packaging ATPase and DNA polymerase in *Megavirales* members**. Protein sequences were aligned using the MUSCLE program with default parameters; all the alignments were conserved, even the columns containing a large fraction of gaps. The maximum-likelihood phylogenetic reconstruction was performed using FastTree. The Faustoviruses cluster with members of *Asfarviridae* and *Poxviridae*. The tree is unrooted. Each *Megavirales* family was represented by a specific color. The values near the branches (green) are bootstrap values.

To determine which *Megavirales* family the Faustovirus is most closely related to, we concatenated the A32-like packaging ATPase and DNA polymerase of representative members of *Megavirales* families. Protein sequences were aligned using the MUSCLE program with default parameters. All the alignments were conserved, even the columns containing a large fraction of gaps. The phylogenetic tree maximum-likelihood phylogenetic reconstruction (**Figure 4**) shows that Faustovirus members are close but different from *Asfarviridae*. These findings are in correlation with the phylogenetic studies conducted by Reteno et al. (2015) This suggests that Faustoviruses represent a new family in the order of *Megavirales*. The gene repertoire of the common ancestor of Faustoviruses and Asfarviridae (named Lucasvirus) was determined by the selection of their core-genes to which we added the common genes of each one with 59 viruses of the *Megavirales* order. Over 40% of the *Asfarviridae* pan-genome takes part in the constitution of the Lucasvirus’ repertoire against only 28.32% of the Faustovirus pan-genome. Supplementary Figure S5 shows the taxonomy of the best matches from the blastp result. We observed that the Lucasvirus and the *Asfarviridae* have fewer good matches with genes from Bacteria (10.97 and 14.86% respectively) than Faustoviruses (30.10%). On the other hand, the percentage of matches with genes from Eukaryota is much higher in Lucasvirus and *Asfarviridae* (40.54 and 40.24% respectively) than in the Faustovirus (23.11%). We observed that the percentage of matches with genes from Viruses and Archaea is almost identical in the three viruses.

### Analysis of Amino Acids and COG Category Distribution

**FIGURE 5:**
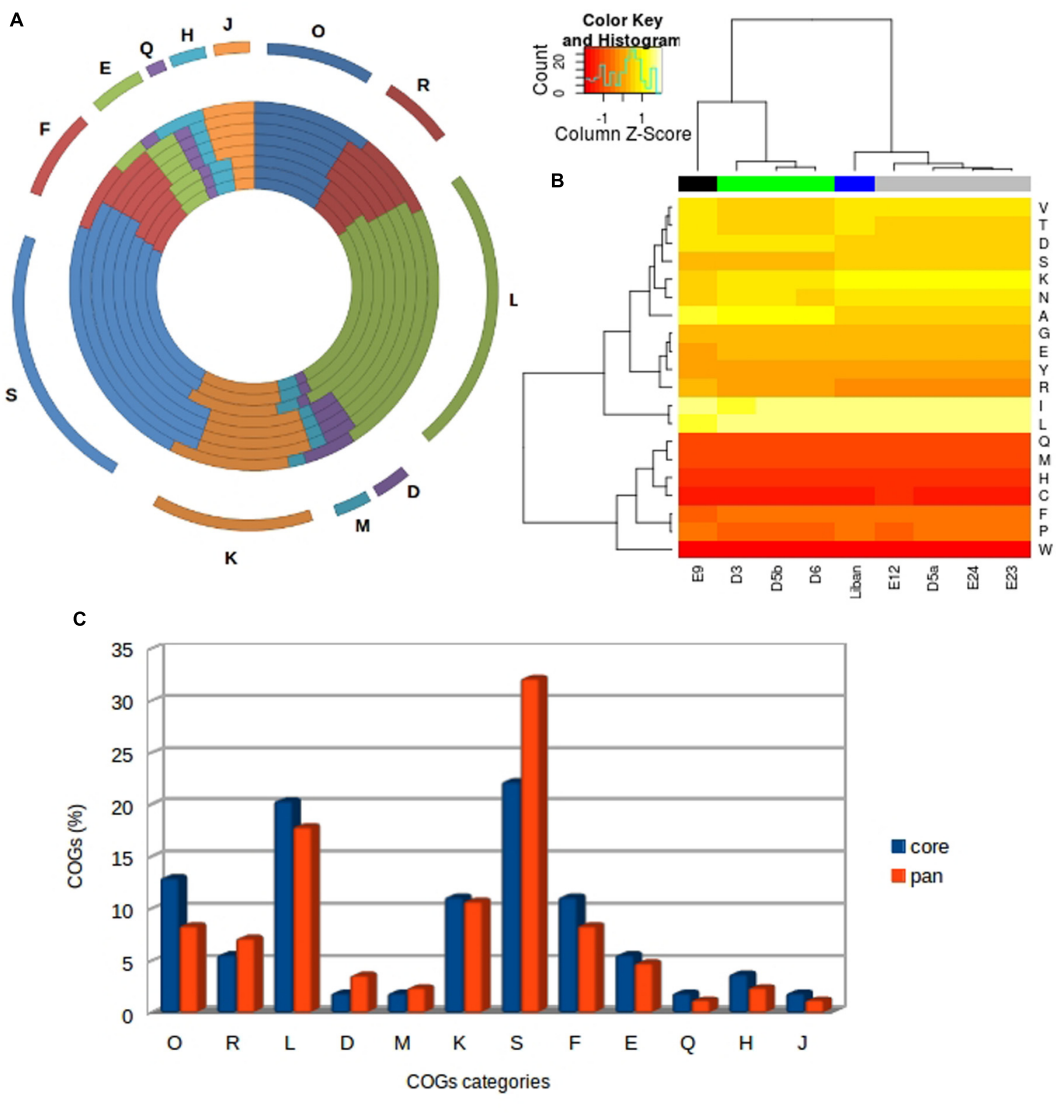
**Analysis of amino acids and COGs category distribution. (A)** Cluster of Orthologs (COG) classification of the families of orthologs. The most abundant families have also been indicated and they are assigned to housekeeping functions. From outer to inner circle: Faustovirus D3, Faustovirus D6, Faustovirus D5b, Faustovirus E12, Faustovirus E24, Faustovirus E23, Faustovirus D5a, Faustovirus Liban and Faustovirus E9. **(B)** Hierarchical clustering heat map representing the variability of Faustoviruses in terms of amino acid composition for nine complete Faustovirus genomes. **(C)** The COG category comparison between the core and the pan-genome of Faustoviruses.

Amino acid composition is very similar among the studied Faustoviruses; however, we noted some differences between members from different lineages (**Figure 5B**). Indeed, we observed that the Faustoviruses of lineage D and E9 are more enriched in alanine, aspartic acid and arginine than other lineages (Supplementary Figure S2). On the other hand, E9 is less enriched in lysine and leucine than other viruses. The viruses of lineage M have a greater portion of asparagine, lysine, serine and valine than other lineages. Some amino acids are considerably more prevalent in all Faustoviruses such as two hydrophobic residues leucine and isoleucine (8.12 and 8.07% average composition respectively). In contrast, tryptophan, cysteine, histidine, methionine, and glutamine (1.04, 1.98, 2.25, 2.63, and 2.87% average composition respectively) are less prevalent. Clustering based on the amino acid composition shows the same topology as the core-gene-based phylogeny.

An average of 15.21% of all Faustovirus genes has an ortholog identified by the Cluster of Orthologous Group (COG) classification (**Figure 5A**). Only 12 among the 25 COGs categories’ were assigned to these genes and over half of them are predicted to be involved in two categories: L (replication, recombination and repair) and S (function unknown). The distribution of COG categories is different among the Faustovirus lineages with the exception of the Q category (secondary metabolite biosynthesis, transport and catabolism), represented by an amino-oxidase protein in all Faustovirus genomes. The viruses of the same lineage have an identical category distribution excluding lineage D, which has a small difference with the L (DNA replication, recombination and repair) COG category. As observed in the amino acid composition, clustering based on COG category distribution shows the same topology as the core-Gene-based phylogeny (Supplementary Figure S3).

### Faustovirus Unique Gene Set

The comparative analyses allowed the identification of an average of 66 unique genes per lineage, mostly represented by hypothetical protein. In fact, the COG classification showed that the majority of these genes have an unknown function. Lineage M has the smallest number of unique genes; indeed, it shares over 94% of its genes with the other lineages. The viruses of lineage D, with their 110 unique genes (27.7% of their gene repertory) show the greatest gene composition diversity. The number of unique genes identified in each virus is more reduced compared to that of the lineages (**Figures 3A,B**). The best matches of the lineages’ unique genes with detectable homologs were mainly with genes from Bacteria (46.15%). The best matches with genes from viruses are represented by four families: *Marseilleviridae* (2.56%), *Mimiviridae* (5.12%), *Asfarviridae* (2.56%), and *Phycodnaviridae* (12.82%), which has the greatest prevalence. Only unique genes of lineages D and E9 have a best match with genes from Archaea. To summarize, the unique genes are mostly composed by ORFans having no match (only 14% have a match while we observed almost 44.92% in the coregenes for example). The genes having a match, are composed by: hypothetical protein (79.48%), MORN variant repeat protein (7.69%), AAA family ATPase, DNA methyltransferase, HNH endonuclease, cell wall anchor protein and the Phage integrase (Supplementary Table S3). The unique genes of the lineage D and E9 are composed only by hypothetical proteins and we found that they are richer in bacterial original genes compared to the coregenes and the pangenome.

## Conclusion

An emerging interest for the giant virus discovery process, genome sequencing and analysis has allowed an expansion of the number of known *Megavirales* members (Boughalmi et al., 2013; Pagnier et al., 2013; Philippe et al., 2013; Saadi et al., 2013; Aherfi et al., 2014; Legendre et al., 2014; Antwerpen et al., 2015; Assis et al., 2015; Scheid, 2015). *Acanthamoeba castellanii* and *A. polyphaga* being the only cellular supports used to isolate these viruses, this approach may neglect a part of the giant virus world. Reteno et al. (2015) using another protist *Vermamoeba* sp. as cell support, have isolated a new giant virus: Faustovirus E12. In this study, we describe and analyze the genome sequences of nine Faustoviruses. The average sequence length of these viruses is 467,592.44 bp (minimum: 455,803 bp and maximum: 491,024 bp), making them the fourth largest *Megavirales* genome after Mimiviruses, Pandoraviruses and *P. sibericum* (Arslan et al., 2011; Philippe et al., 2013; Antwerpen et al., 2015; Scheid, 2015). Faustovirus genomes displayed an average G+C content of 37.14% (ranging from 36.22 to 39.59%), which is close to the G+C content range of the *Asfarviridae* genomes (38%) (Wan et al., 2013). The Faustoviruses’ pan-genome is still open. Indeed, the clustering identified 872 protein clusters while the deduced mathematical function predicted that the Faustoviruses’ pan-genome probably surpasses 1000 genes. On the other hand, the core-genome appears to consolidate the surrounding of 207 genes. The number of core-genes in each genome is constant, except for the duplication of five Faustovirus Liban genes. Research of duplicated genes in remaining clusters has highlighted five duplications in Faustovirus E9 and eight other duplications in Faustovirus Liban. There are no duplicated genes, which implies an absence of paralogs, for other Faustoviruses were observed. This finding suggests that the genomic diversity of Faustoviruses is essentially due to gene deletion or acquisition and timely to minor duplications. A comparison between the COG classification of the pan and core-genome showed that the last one contains a majority of genes involved in various housekeeping functions (**Figure 5C**). We identified especially those related to: (i) nucleotide, coenzyme and amino acid metabolism and associated transport activities; (ii) translation, ribosomal structure and biogenesis and transcription replication, recombination and repair. This is in agreement with the conserved developmental cycle and the intracellular lifestyle of these viruses. The core-genome displays a larger number of genes with detectable homologs than the pan-genome. The proportion of best matches from viruses in general and from *Asfarviridae* in particular is greater in the core-genome than in the pan-genome. On the basis of these observations, we may assume that the Faustoviruses share a common origin with *Asfarviridae*. The phylogenetic analysis is simply a confirmation of that fact, indeed based on the phylogenetic tree the Faustoviruses are close to *Asfarviridae* but still too different to establish a same family. The matches with genes from Bacteria and Eukaryota were similar between Lucasvirus and *Asfarviridae* while we observed a significant gap between Lucasvirus and Faustovirus. These results suggest that Faustoviruses have a precocious divergence from Lucasvirus compared to *Asfarviridae*. The low percentage of Faustovirus pan-genomes included in the Lucasvirus gene repertoire indicates an enrichment of Faustovirus with genes from horizontal gene transfer (HGT).

We observed more best matches from Bacteria and *Phycodnaviridae* in the pan-genome than in the core-genome. Their proportion is significantly higher in the unique genes. This may be due to an interaction occurring after the separation of the lineages. The core-gene-based phylogeny of Faustoviruses study identified four lineages and these results were confirmed by the analysis of amino acids and COGs category distribution. Indeed, lineages M and L share a huge number of genes (9.86% of the pan-genome) as well as lineages E9 and D (6.42%) which refer to the two main clusters of the core-gene-based phylogeny. The reproducibility of the clustering in four lineages suggests their earlier separation. The study of the unique gene distribution shows greater prevalence when we focus on lineages than on an individual virus, suggesting that the inter-lineage diversity is much greater than the intra-lineage diversity.

In summary, whole genome sequencing and pan-genome analysis of Faustoviruses in this study revealed that the representative members of this family were separated into four lineages and the core-genome represents one quarter of their pan-genome. These findings point to a great diversity of gene composition mainly explained by gene deletion or acquisition and some exceptions for gene duplications (Faustovirus E9 and Faustovirus Liban). Regarding the separation on four lineages, we observed that the clustering was confirmed by several analyses; this would suggest their earlier separation. The high proportion of best matches from Bacteria and *Phycodnaviridae* on the pan-genome and unique genes may be due to an interaction occurring after the separation of the lineages. The Faustoviruses’ pan-genome is described as an open pan-genome; its enrichment via the discovery of new Faustoviruses is required to better grasp the full genomic diversity of this family.

## Author Contributions

SB wrote the manuscript and did bioinformatic analysis, DGIR isolated Faustoviruses and did their presumptive identification, VB isolated Faustovirus, NL did genome sequencing, DR reviewed the manuscript and did several corrections, BL designed the study and wrote the manuscript.

## Conflict of Interest Statement

The authors declare that the research was conducted in the absence of any commercial or financial relationships that could be construed as a potential conflict of interest.
