# Supplementary material for: Faustoviruses: Comparative Genomics of New Megavirales Family Members
Source: Front Microbiol. 2016 Feb 5;7:3. doi: 10.3389/fmicb.2016.00003 (PMC4742530; doi:10.3389/fmicb.2016.00003)
Supplement: Supplementary file 2 [file Table_2.PDF]

Table S2 : The Clusters of Orthologous Groups (COGs) repartition in the pangenes, coregenes and the unique genes.

	Pangenes	Coregenes	Unique genes
**Cellular processes and signaling**	**12**	**9**	**2**
Cell cycle control and cell division	3	1	1
Cell wall/membrane/envelope biogenesis	2	1	1
Post-translational modification	7	7	0
Defense mechanisms	0	0	0
**Information storage and processing**	**25**	**18**	**0**
Translation, ribosomal structure and biogenesis	1	1	0
Transcription	9	6	0
Replication, recombination and repair	15	11	0
**Metabolism**	**11**	**9**	**0**
Amino acid transport and metabolism	3	2	0
Nucleotide transport and metabolism	5	4	0
Carbohydrate transport and metabolism	0	0	0
Coenzyme transport and metabolism	2	2	0
Lipid transport and metabolism	0	0	0
Secondary metabolites biosynthesis, transport, and catabolism	1	1	0
**Poorly characterized**	**29**	**11**	**2**
General function prediction only	6	3	0
Function unknown	23	8	2
**No Clusters of Orthologous Groups (COGs)**	**795**	**160**	**178**
