# Supplementary material for: Faustoviruses: Comparative Genomics of New Megavirales Family Members
Source: Front Microbiol. 2016 Feb 5;7:3. doi: 10.3389/fmicb.2016.00003 (PMC4742530; doi:10.3389/fmicb.2016.00003)
Supplement: Supplementary file 3 [file Table_3.PDF]

Table S3. Repartition of the unique genes in the pangenome and in the different Faustovirus lineages.

	Proteins with best match (%)	Best match repartition				Proteins function
		Bacteria (%)	*Virus (%)*	*Eukaryota (%)*	*Archaea (%)*	
All unique genes (267 genes)	14.6	46.15	30.76	17.94	5.12	hypothetical protein<br>putative AAA family ATPase<br>DNA methyltransferase<br>HNH endonuclease<br>cell wall anchor protein<br>Phage integrase<br>MORN variant repeat protein
D unique genes (110 genes)	15.45	35.29	29.41	29.41	5.88	hypothetical protein<br>putative AAA family ATPase<br>DNA methyltransferase<br>HNH endonuclease<br>cell wall anchor protein<br>Phage integrase<br>MORN variant repeat protein
M unique genes (19 genes)	42.10	62.5	37.5	0	0	hypothetical protein<br>Putative structural protein<br>MORN repeat containing protein
L unique genes (47 genes)	10.63	80	20	0	0	hypothetical protein
E9 unique genes (91 genes)	9.89	33.33	33.33	22.22	11.11	hypothetical protein
