# Supplementary material for: Faustoviruses: Comparative Genomics of New Megavirales Family Members
Source: Front Microbiol. 2016 Feb 5;7:3. doi: 10.3389/fmicb.2016.00003 (PMC4742530; doi:10.3389/fmicb.2016.00003)
Supplement: Supplementary file 4 [file Data_Sheet_1.PDF]

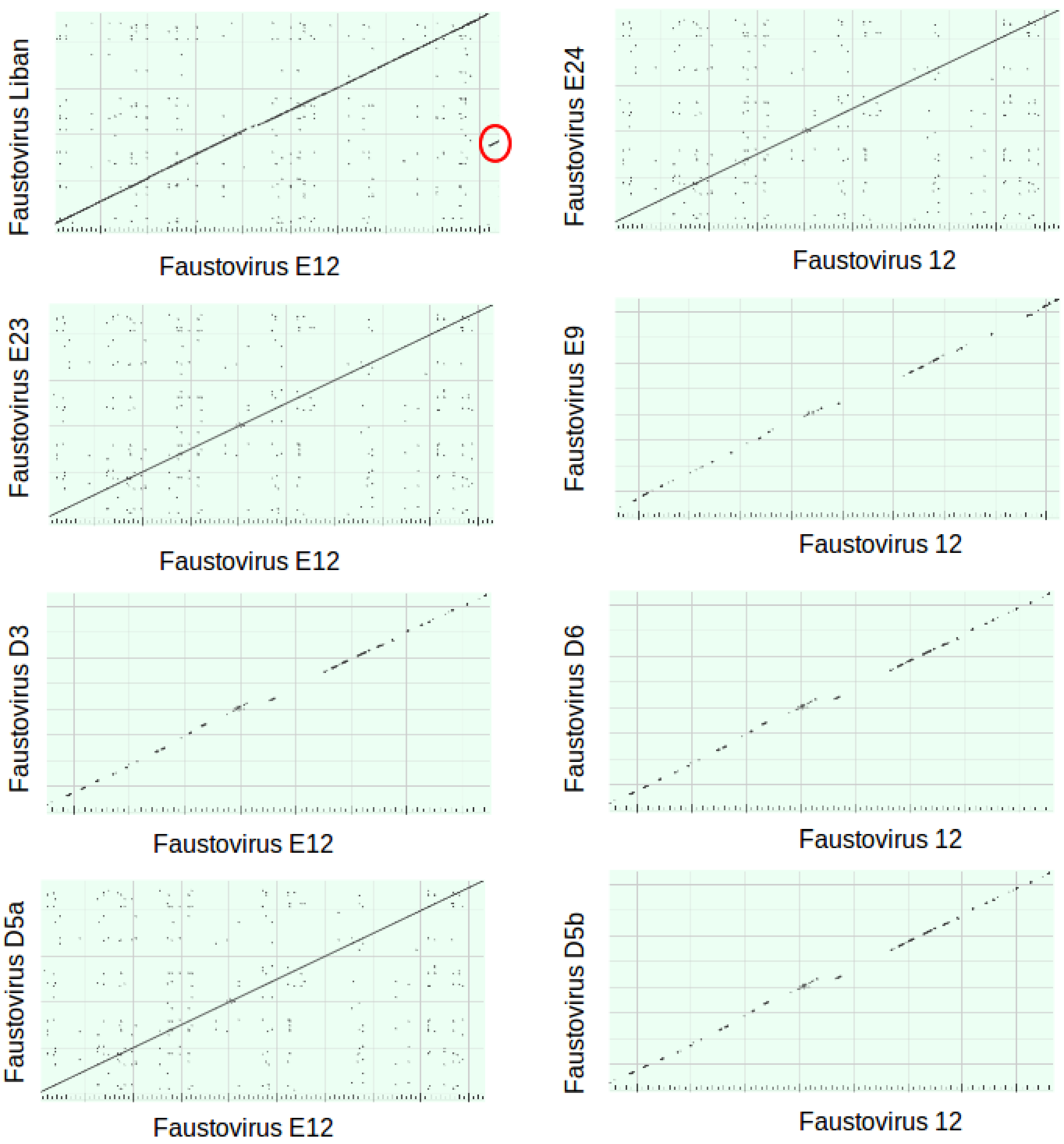

Figure S1 : **Synteny of Faustovirus genomes**. BLAST dot plots showing the high degree of conservation of gene order between the Faustovirus E12 genome and the others genomes of the lineage M. A lack of synteny within the Faustovirus E12 and the members of the other lineages was observed. Complete genome sequences were used. The duplication were observed only with the Faustovirus Liban (showed in red circle).

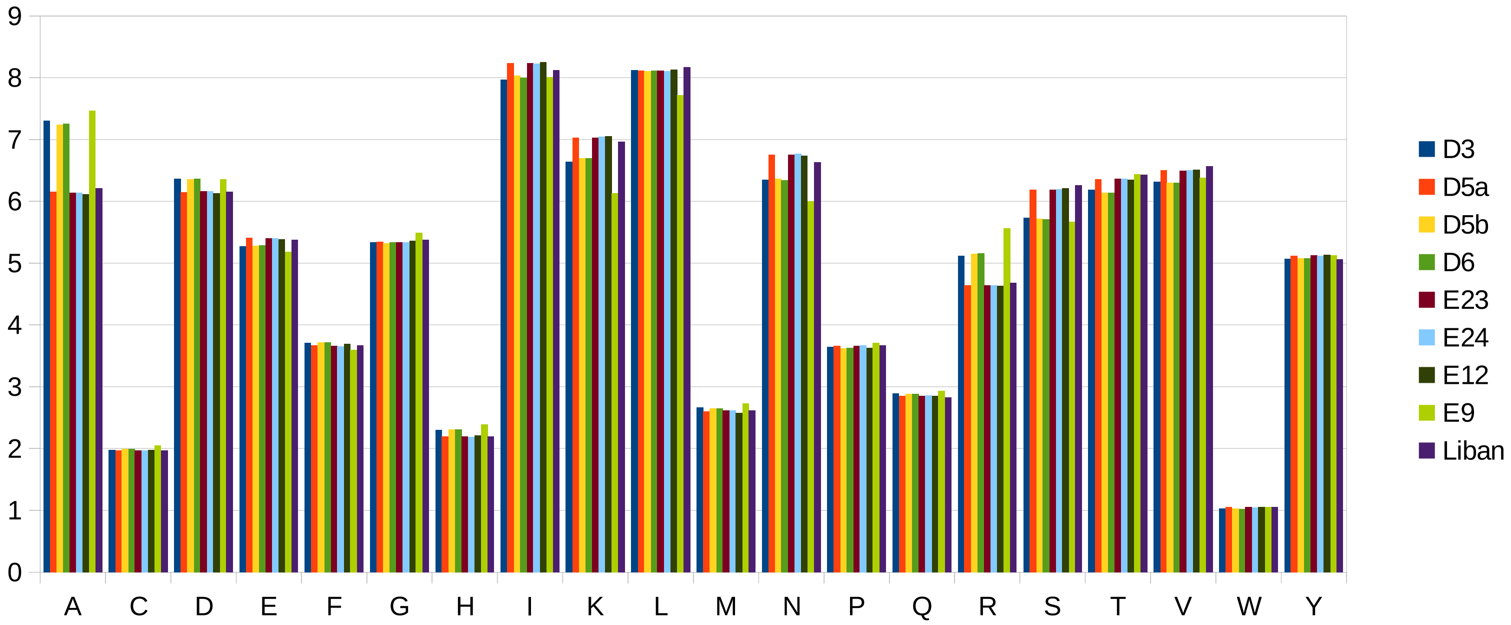

Figure S2: The amino acids comparison between the nine Faustovirus genomes.

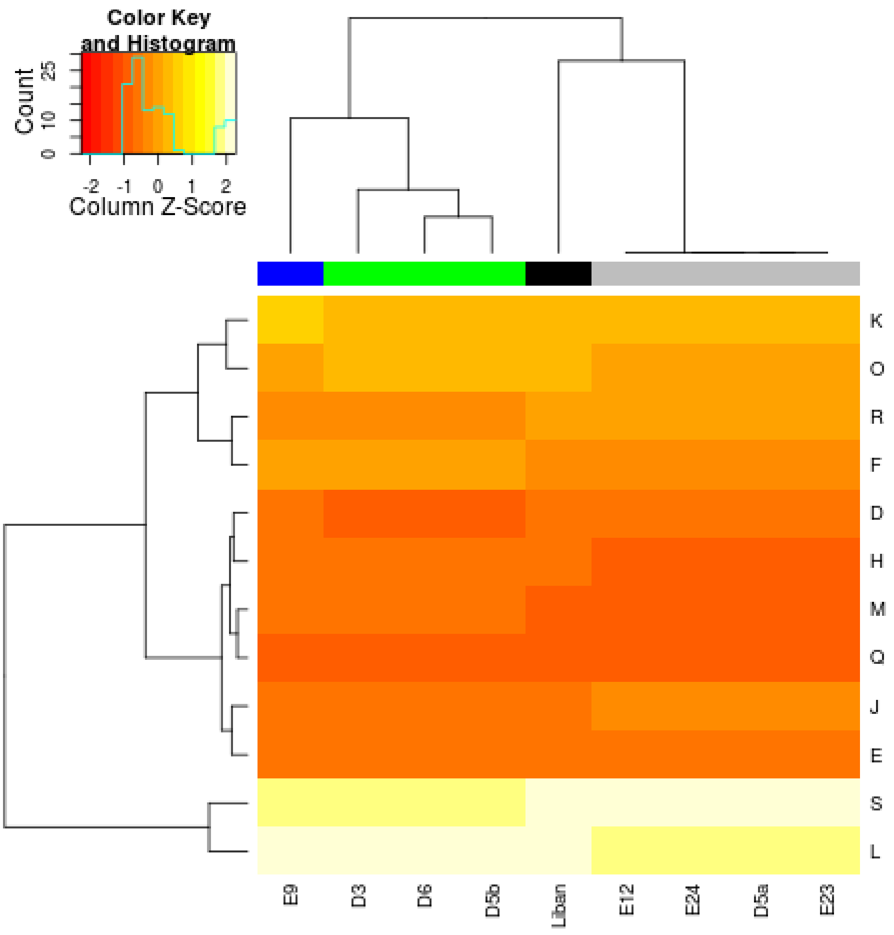

Figure 3S: Hierarchical clustering heatmap representing the variability of Faustoviruses in terms of COGs categories composition for nine complete genomes of Faustovirus.

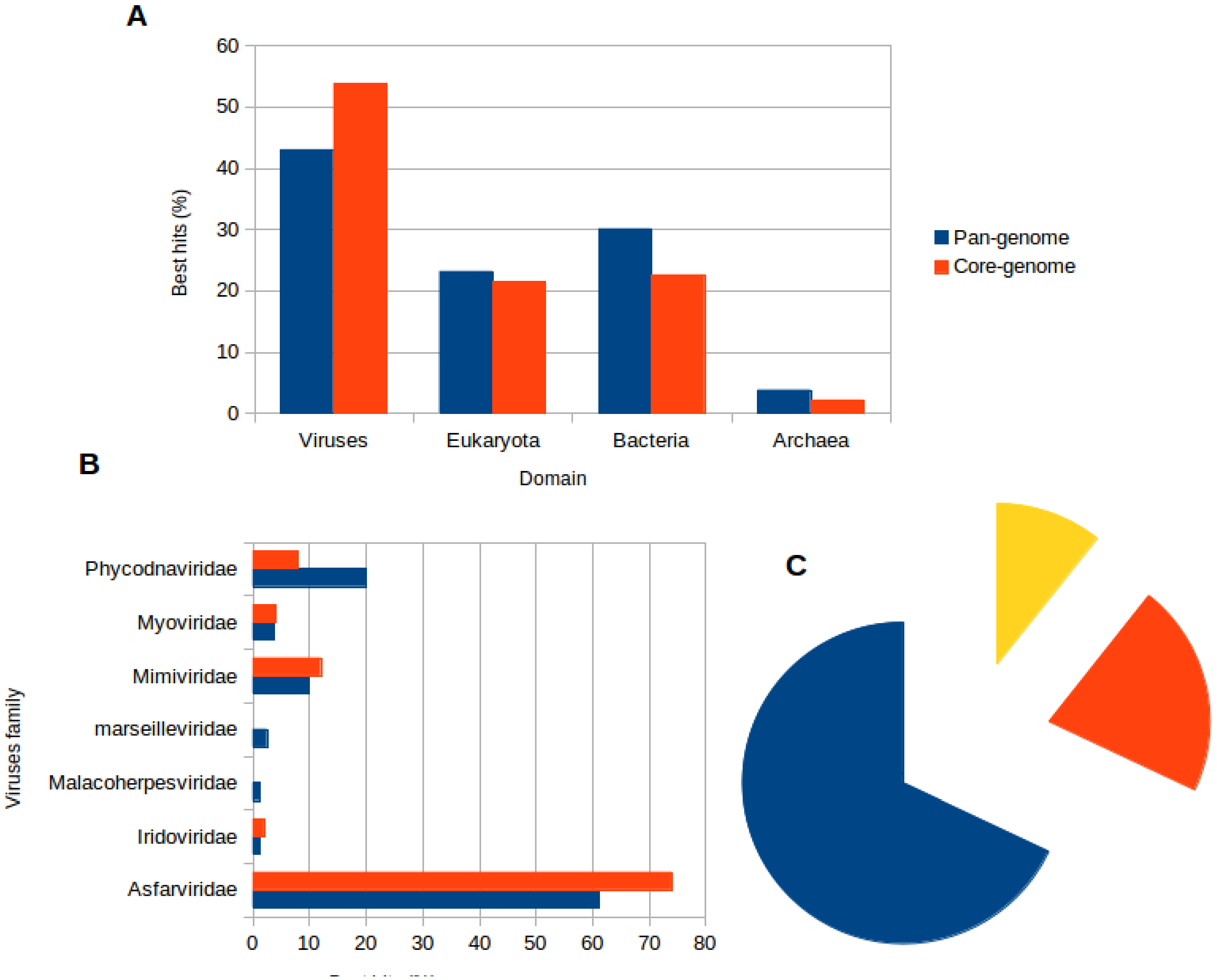

Figure S4: Taxonomy of the best hits from the Blast result. A: comparison of the domain partition between the core and pan-genome. **B**: comparison of the Viruses partition between the core and pan-genome. **C**: proportion of the pan-genome blast hits. CDSs with no blast result were represented in blue, the core-genes with blast result in yellow and the remaining genes with a blast result in red.

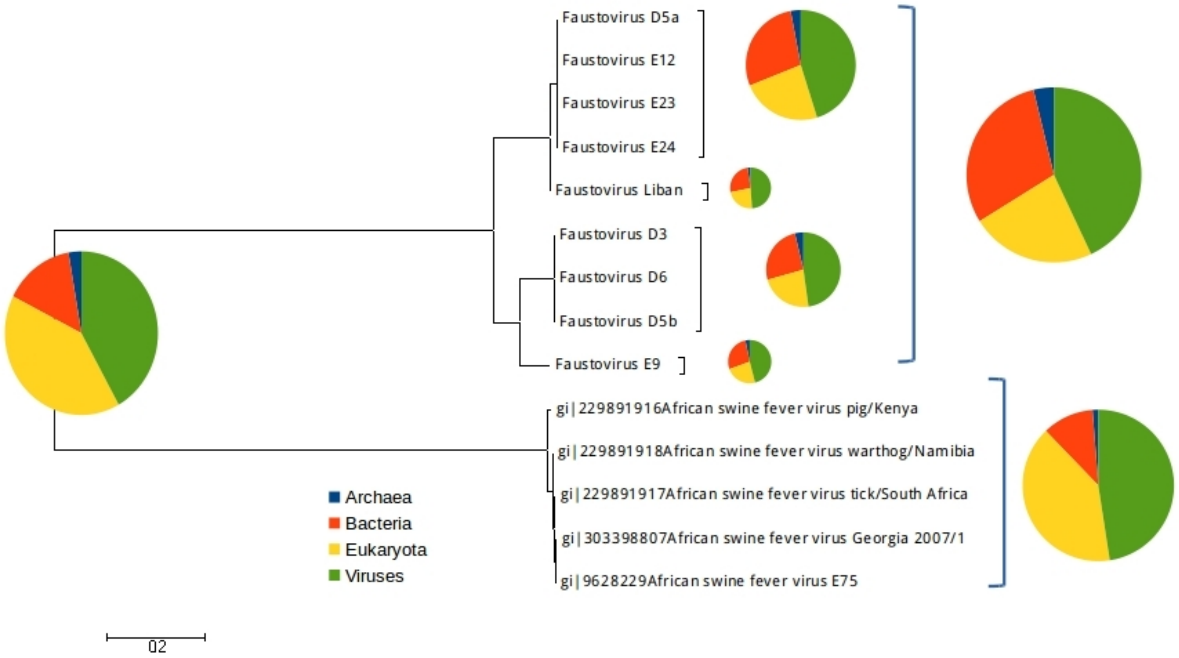

Figure S5 : Taxonomy of the best hits from the Blast result of Faustovirus, Asfarviridae and their common ancestor.
